# Identification of functional near-infrared spectroscopy for older adults with mild cognitive impairment: a systematic review

**DOI:** 10.3389/fnagi.2025.1492800

**Published:** 2025-04-09

**Authors:** Bo Yang, Xia Deng, Xianfeng Qu, Yingjie Li, Lei Guo, Nengwei Yu

**Affiliations:** ^1^Department of Center for Psychosomatic Medicine, Sichuan Provincial Center for Mental Health, Sichuan Provincial People’s Hospital, University of Electronic Science and Technology of China, Chengdu, China; ^2^Department of Radiology, Sichuan Provincial People’s Hospital, University of Electronic Science and Technology of China, Chengdu, China; ^3^Department of Neurology, Sichuan Provincial People’s Hospital, University of Electronic Science and Technology of China, Chengdu, China; ^4^Department of Neurology, Xindu District People’s Hospital of Chengdu, Chengdu, China

**Keywords:** cerebral hemodynamics, cerebral oxygenation, functional near-infrared spectroscopy, mild cognitive impairment, systematic review

## Abstract

**Objective:**

Mild cognitive impairment (MCI), a common state of cognitive impairment without significant impairment in daily functioning among older adults, is mainly identified using various neuropsychological tests, clinical interviews, and collateral history with some subjective interferences. This systematic review aimed to investigate the functional near-infrared spectroscopy (fNIRS) features of older adults with MCI compared with those with normal cognitive function to assist in the diagnosis of MCI.

**Methods:**

A literature search was conducted in electronic databases, including PubMed, Web of Science, Embase, and Cochrane Library, up to June 15, 2024. The data on article information (first author and year of publication), participant characteristics, task paradigms, regions of interest (ROIs), fNIRS device attributes, and results related to cerebral oxygenation and hemodynamics were extracted.

**Results:**

Finally, 34 relevant studies were identified, involving 1033 patients with MCI and 1107 age-, sex-, and education-matched controls with normal cognitive function. We found that the studies frequently used working memory–related task paradigms and resting-state measurements. Also, the prefrontal cortex was a primary ROI, and the changes in oxygenated hemoglobin concentration were the most basic research attributes used to derive measures such as functional connectivity (FC), FC variability, slope, and other parameters. However, ROI activation levels differed inconsistently between patients with MCI and individuals with normal cognition across studies. In general, the activation levels in the ROI of MCI patients may be higher than, lower than, or comparable to those in the normal control group.

**Conclusion:**

Research on fNIRS in elderly patients with MCI aims to provide an objective marker for MCI diagnosis. The current findings are mixed. However, these differences can be partly explained with the theoretical support from the interaction of cognitive load theory and scaffolding theory of aging and cognition, taking into account factors such as unspecified MCI subtypes, task difficulty, task design, monitoring duration, and population characteristics. Therefore, future studies should consider definite MCI subtypes, strict and well-designed paradigms, long-term monitoring, and large sample sizes to obtain the most consistent results, thereby providing objective references for the clinical diagnosis of MCI in elderly patients.

## 1 Introduction

The healthy aging process usually causes cognitive changes in healthy older adults; also, the cognitive decline in patients with mild cognitive impairment (MCI) is more severe than expected for their age (Tuokko and Mcdowell, 2006). Generally, MCI is defined as an intermediate state of cognitive impairment between healthy aging and dementia (Albert et al., 2011). Epidemiological studies show that MCI is highly prevalent among individuals aged more than 65 years, affecting about 10%–20% of this population (Di Carlo et al., 2007). Additionally, according to Mayo MCI criteria: (i) memory complaint, preferably corroborated by an informant, (ii) objective memory impairment for age, (iii) relatively preserved general cognition for age, (iv) essentially intact activities of daily living, and (v) not demented. about 12% of patients with MCI progress to Alzheimer’s disease (AD) annually (Petersen, 2004). And Petersen (2004) proposed the amnestic MCI criterion: (i) Memory complaint usually corroborated by an informant, (ii) Objective memory impairment for age, (iii) Essentially preserved general cognitive function largely intact functional activities, (iv) Not demented. In Diagnostic and Statistical Manual of Mental Disorders, Fifth Edition, memory impairment was replaced by multiple cognitive impairment categories (complex attention, executive function, learning and memory, language, perception-action or social cognition). Early identification and intervention for elderly individuals in the prodromal stage of AD are crucial prophylactic–therapeutic measures. At present, the diagnosis of MCI primarily relies on neuropsychological tests, clinical interviews, and collateral history. But because of wide variation in neuropsychological tests reported specificity and sensitivity (De Roeck et al., 2019). And these tests are highly influenced by motivation and attention (Brown, 2015). We are exploring methods to monitor brain function to assist in the diagnosis of MCI.

Brain function tests can be generally classified into two categories: electrophysiological examinations (electroencephalogram and magnetoencephalogram) and blood oxygen examinations [positron emission computed tomography, functional magnetic resonance imaging, and functional near-infrared spectroscopy (fNIRS)]. Among these, fNIRS is a noninvasive neuroimaging technology with a unique anti-interference capability, allowing it to assess brain function levels even during speech or movement by measuring changes in the concentrations of local oxygenated and deoxygenated hemoglobin (HbO2 and HbR) (Scholkmann et al., 2014). The local oxygen metabolism rate and cerebral blood flow rate increase when the cerebral cortex is active to provide the energy required for neuronal activity (Scholkmann et al., 2014). This is defined as functional brain congestion and neurovascular coupling (Nippert et al., 2018). Finally, in the active brain regions, HbO2 levels ultimately increase, whereas HbR levels decrease because of the increase in the concentration of local oxygenated blood. In addition, fNIRS has the following advantages: portable, low cost, convenient operation and low demand for the subjects. Thus, fNIRS is suitable for assessing the brain function in elderly patients with MCI.

Compared to healthy younger populations, current fNIRS studies in cognitively healthy older adults reveal the following characteristics: (1) Compensatory overactivation in the prefrontal cortex (Heinzel et al., 2013); (2) Reduced resting-state functional connectivity (Yeung and Chan, 2020); (3) Delayed hemodynamic responses (Schroeter et al., 2002). These changes reflect adaptive neurovascular remodeling during aging. In contrast, individuals with dementia exhibit generally suppressed hemodynamic responses on fNIRS, including more pronounced reductions in resting-state connectivity and hypoactivation (Butters et al., 2023). However, findings in MCI patients remain inconsistent, potentially demonstrating both hypoactivation and compensatory hyperactivation patterns (Butters et al., 2023). In this systematic review, we discussed the fNIRS features of older adults with MCI compared with those with normal cognitive function, aiming to provide a new marker for assessing cognitive function in these patients.

## 2 Materials and methods

The guidelines from the Preferred Reporting Items for Systematic Reviews and Meta-analysis statements were referenced in this study (Page et al., 2021).

### 2.1 Search strategy

The literature search was conducted in electronic databases, including PubMed, Web of Science, Embase, and Cochrane Library, up to June 15, 2024. The following two groups of keywords were used: (1) mild cognitive impairment, mild cognitive dysfunction, mild cognitive disorder, MCI, and (2) fNIRS, functional near-infrared spectroscopy, near-infrared spectroscopy, and NIRS. The terms related to keywords and medical subject headings were used in this search. Also, relevant professional journals and reference lists of publications were searched when necessary. The search algorithm for each database is provided in the Supplementary material. Only articles with full-text availability in English were included.

### 2.2 Study selection

The PICO framework was used to formulate the inclusion criteria, which were as follows: (1) Population (P): older adult participants with MCI (aged more than 60 years; considered a pre-dementia or pre-AD stage; objective evidence of cognitive impairment came from standardized neuropsychological tests; no significant impairment in daily life function; cognitive impairment does not only occur in delirium situations and cannot be better explained by another mental illness; no other neurological disorders; amnestic MCI and non-amnestic MCI included according to Petersen’s criteria.); (2) Intervention (I): various task paradigms combined with fNIRS; (3) Comparison (C): age-, sex-, and education-matched healthy population with normal cognitive function; and (4) Outcome (O): studies used fNIRS to quantify changes in hemoglobin concentration in brain regions of interest (ROIs).

Duplicate studies were removed using Endnote X9 (Clarivate Analytics, MA, USA), and unrecognized repetitions were manually screened (DX and QXF). Then, the titles, abstracts, and full texts of the remaining studies were screened by two independent reviewers (GL and LYJ) to determine eligibility. The exclusion criteria were as follows: cognitive impairment consistent with dementia; MCI due to other causes, such as trauma and stroke; and no monitoring of the cerebral hemodynamic response during tasks. Conference proceedings, review articles, and case reports were also excluded. Any disagreements on eligibility were resolved through discussion with a third reviewer (YNW).

### 2.3 Data extraction

Three reviewers (YB, DX, and QXF) completed the data extraction task independently using a preliminarily developed extraction sheet. The following data were retrieved from included studies: (1) article information: first author and year of publication; (2) participant characteristics: sample size, age, sex, educational level, and cognition level; (3) task paradigm; (4) ROI; (5) fNIRS device attributes: manufacturer, distance from the transmitter to the detector, wavelength, and sampling frequency; (6) results related to cerebral oxygenation and hemodynamics. For incomplete or unclear data, the corresponding author of the study was contacted by email. The two researchers (GL and LYJ) compared the extracted data for accuracy, and any discrepancies were resolved through discussion with a third reviewer (YNW).

### 2.4 Quality assessment

The quality of the included studies was assessed independently by two reviewers (LYJ and GL) using the Newcastle–Ottawa Scale (NOS) (Stang, 2010). The NOS consists of three domains: selection of study subjects, comparability, and outcome measurement, with eight items. The evaluation was based on a scoring system, with a maximum of nine points. The higher scores suggested a higher quality of the study. The disagreements about the quality were also resolved through discussion with the third independent reviewer (YNW). The results of this assessment are provided in the Table 1.

**TABLE 1:** The Newcastle-Ottawa Scale.

study	Selection				Comparability	Exposure			Quality score
	**Is the case definition adequate**	**Representativeness of the cases**	**Selection of controls**	**Definition of controls**	**Comparability of cases and controls on the basis of the design or analysis**	**Ascertainment of exposure**	**Same method of ascertainment for cases and controls**	**Non-response rate**	
Bu et al., 2019	*	*	*	*	**	*	*	*	**9**
Canario et al., 2022	*	*	*	*	**	*	*	*	**9**
Ghafoor et al., 2019	*	*	*	*		*	*	*	**7**
Haberstumpf et al., 2022	*	*	*	*	*	*	*	*	**8**
Ho et al., 2022	*	*	*	*	*		*	*	**7**
Kato et al., 2017	*	*		*		*	*	*	**6**
Katzorke et al., 2018	*	*	*	*	*	*	*	*	**8**
Khan et al., 2022	*	*	*	*	*	*	*	*	**8**
Kim et al., 2021	*	*	*	*	*	*	*	*	**8**
Li R. et al., 2018	*	*	*	*	*	*	*	*	**8**
Li X. et al., 2018	*	*	*	*	*	*	*	*	**8**
Li et al., 2020	*	*	*	*	**	*	*	*	**9**
Niu et al., 2019	*	*	*	*	**	*	*	*	**9**
Liu et al., 2023	*	*	*	*	*	*	*	*	**8**
Nakamura et al., 2021	*	*	*	*	*	*	*	*	**8**
Nguyen et al., 2019	*		*			*	*	*	**5**
Baik et al., 2022	*	*	*	*	**	*	*	*	**9**
Takahashi et al., 2022	*	*	*	*	*	*	*	*	**8**
Talamonti et al., 2022	*	*	*	*	*	*	*	*	**8**
Ung et al., 2020	*	*	*	*	*	*	*	*	**8**
Vermeij et al., 2017	*	*	*	*	*	*	*	*	**8**
Xu et al., 2024	*	*	*	*		*	*	*	**7**
Yang et al., 2019	*	*	*	*	*	*	*	*	**8**
Yang et al., 2020	*	*	*	*	*	*	*	*	**8**
Yang and Hong, 2021	*	*	*	*	*	*	*	*	**8**
Yap et al., 2017	*	*	*	*	*	*	*	*	**8**
Yeung et al., 2016a	*	*	*	*	*	*	*	*	**8**
Yeung et al., 2016b	*	*	*	*		*	*	*	**7**
Yoo and Hong, 2019	*		*			*	*		**4**
Yoon et al., 2019	*		*			*	*		**4**
Yu et al., 2020	*	*		*		*	*	*	**6**
Zeller et al., 2019	*	*	*	*		*	*	*	**7**
Zhang et al., 2022	*	*	*	*		*	*	*	**7**
Zhang et al., 2023	*	*	*	*		*	*	*	**7**

* Indicated scores in Newcastle-Ottawa Scale.

### 2.5 Presentation of results

Due to the heterogeneity of the clinical populations, methods used, and data presented, meta-analysis was not possible. The results were synthesized into a brief narrative review, focusing on the differences in various fNIRS findings between elderly patients with MCI and those with normal cognitive function.

## 3 Results

### 3.1 Study selection

**FIGURE 1:**
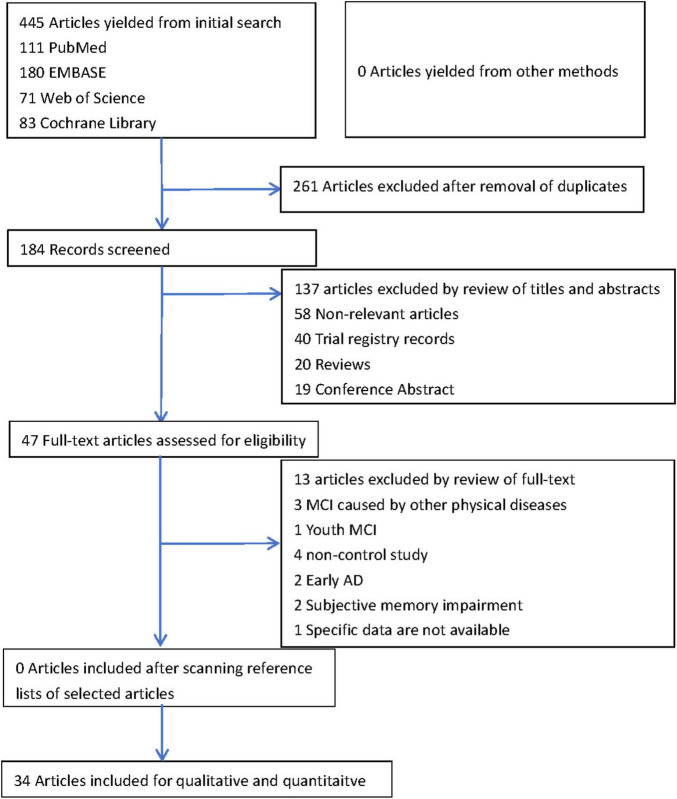
The flow chart of selection process of included records.

We identified 445 records: PubMed (111), Embase (180), Web of Science (71), and Cochrane Library (83). A total of 261 duplicates were excluded, and 184 records were evaluated by titles and abstracts. Then, 13 studies were excluded, including 58 non-relevant studies, 40 trial registry records, 20 reviews, and 19 conference abstracts. Further, we reviewed the full texts of the remaining 47 studies. We found three studies on MCI caused by other diseases (tumor and chronic obstructive pulmonary disease) and five focusing on MCI in young adults, early AD, and subjective memory impairment, which were unsuitable for inclusion in the study. In addition, four non-control studies and one study without specific data were excluded. No additional articles were included using other methods or by scanning reference lists of selected articles. Finally, 34 studies investigated the fNIRS differences between older adults and patients with MCI were included. A flow chart presenting the selection of included records is displayed in Figure 1.

### 3.2 Study characteristics

#### 3.2.1 Demographic characteristics

All included studies were non-randomized controlled trials involving 1033 elderly patients with MCI (recorded male/female: 450/511; amnestic MCI 288 in studies documenting subtypes of MCI) and 1107 age-, sex-, and education-matched normal cognitive old individuals. The sample size of MCI varied from 8 to 98 cases, and the average age of the study population ranged from 61.58 to 78.1 years. The cognitive function scales used in the studies included Mini-Mental State Examination (average score 21.4–28.9), Montreal Cognitive Assessment (average score 17.3–27.0), and Clinical Dementia Rating (average score 0.3–0.5). A few studies used the Auditory Verbal Learning Test and Hasegama’s Dementia Scale-Revised (HDS-R). The scores of all research participants met the MCI criteria. Further details on article information, fNIRS device attributes, quality assessment, and qualitative synthesis are shown in Table 2.

**TABLE 2:** The details of all included studies.

Study	MCI Population				MCI cognitive function	MCI diagnostic criteria	Task paradigm	ROI	fNIRS device attributes	Results of fNIRS compared to control
	***N***	**Age(y)**	**Sex(M:F)**	**educational level(y)**						
Bu et al., 2019	26	69.3 ± 3.6	13:13	NA	MMSE :26.2 ± 1.7 MoCA :23.9 ± 1.3	Neuropsychological tests support daily living ability preserved and no other neurological and psychiatric disorders	Resting state	PFC, MC, OC	Device:Nirsmart (Danyang Huichuang Medical Equipment Co., Ltd., China) Wavelength:780, 808 and 850 nm Channel:14 Sampling rate:10 Hz	EC among PFC, MC and OC ↓
Canario et al., 2022	29 (amnestic MCI)	70.9 ± 10.0	16:13	11.3 ± 4.6	AVLT-I:6.2 ± 2.7 AVLT-D:4.4 ± 3.9 AVLT-R:8.2 ± 3.6 MMSE:22.2 ± 4.4 MoCA:19.7 ± 4.9	Neuropsychological tests support daily living ability preserved and no other neurological and psychiatric disorders	Resting state	PFC, PC	Device:CW6 (TechEn Inc.; MA, USA) Channel:46	FC among PFC and PC no difference
Ghafoor et al., 2019	12	61.6 ± 6.6	00:12	NA	MoCA-K:19.3 ± 2.2	Neuropsychological tests support daily living ability preserved and no other neurological and psychiatric disorders	Resting state	PFC	Device:NIR Scout (NIRx Medizintechnik GmbH; Berlin, Germany) Wavelength:780 and 850 nm Channel:20 Sampling rate:7.81 Hz	PFC FC↓
Haberstumpf et al., 2022	59	74.1 ± 1.6	09:26	9.6 ± 3.0	MMSE:28.9 ± 1.2	EADC (Portet et al., 2006)	Clock angle discrimination task	PC	Device:ETG-4000 (Hitachi Medical Corporation, Tokyo, Japan) Wavelength:695 and 830 nm Channel:22 Sampling rate:10 Hz	PC △oxyHb and lateralization↓
Ho et al., 2022	50	75.8 ± 3.9	07:17	10.6 ± 5.2	MMSE:26.0 ± 3.2	NINCDS-ADRDA (McKhann et al., 1984); NIA-AA (Jack et al., 2011), IWG (Cummings et al., 2013)	Resting state	PFC	NA	PFC △oxyHb no difference
Kato et al., 2017	LSMG:65 HSMG:33	LSMG:75.8 ± 6.2 HSMG:78.1 ± 6.8	LSMG:21:44 HSMG:12:21	NA	HDS-R:LSMG:26.4 ± 1.5 HSMG:23.5 ± 1.8 MMSE:LSMG:27.1 ± 1.3 HSMG:25.4 ± 1.3 CDR:LSMG:0.3 ± 0.2 HSMG:0.5 ± 0.2 VSRAD:LSMG:1.1 ± 0.5 HSMG:1.8 ± 0.7	neuropsychological tests support daily living ability preserved and no other neurological and psychiatric disorders	Shiritori task	PFC, parietal association area	Device:ETG-4000 (Hitachi, Tokyo, Japan) Channel:44	LSMG: bilateral PFC ΔoxyHb↓
Katzorke et al., 2018	55	74.0 ± 1.6	21:34	9.4 ± 2.9	DemTect:14.6 ± 2.3 MMST:28.8 ± 1.3	neuropsychological tests support daily living ability preserved and no other neurological and psychiatric disorders	VFT	PFC, TC	Device:ETG-4000, Hitachi Medical Corporation, Tokyo, Japan Wavelength:695 and 830 nm Channel:52 Sampling rate:10 Hz	PFC and TC reduced hemoglobin (HbR)↓
Khan et al., 2022	11	61.6 ± 6.6	00:11	NA	MoCA-K < 22	neuropsychological tests support daily living ability preserved and no other neurological and psychiatric disorders	Visuospatial working memory task	PFC	Device:NIRScout(NIRx Medical Technologies, New York, NY, USA) Wavelength:760 and 850 nm Channel:204 Sampling rate:7.81 Hz	Bilateral PFC △oxyHb↓
Kim et al., 2021	11	78.2 ± 5.4	02:09	3.2 ± 4.8	K-MMSE:21.4 ± 4.7	neuropsychological tests support daily living ability preserved and no other neurological and psychiatric disorders	Delayed match to sample task, digit span test	PFC	Device:NIRSIT, OBELAB, Seoul, South Korea Wavelength:780 and 850 nm Channel:20 Sampling rate:8.138 Hz	PFC △oxyHb ↑
Li R. et al., 2018	9	70.3 ± 5.4	06:03	NA	MMSE:26 ± 2.2	neuropsychological tests support daily living ability preserved and no other neurological and psychiatric disorders	Digit verbal span task	PFC and PC	Device:NIRScout system (NIRx Medizintechnik GmbH, Germany) Wavelength:760 and 850 nm Channel:46 Sampling rate:3.91 Hz	Bilateral PFC and PC △oxyHb no difference bilateral PFC △oxyHb slope↓
Li X. et al., 2018	27 (amnestic MCI)	70.3 ± 8.3	14:13	10.7 ± 5.0	MMSE:23.7 ± 4.7 MOCA:19.2 ± 5.2 AVLT-I:6.3 ± 2.6 AVLT-D: 4.4 ± 3.9 AVLT-R:8.1 ± 3.6	Petersen’s (Petersen, 2004)	Resting state	PFC, TC, PC, OC.	Device:CW6 (TechEn Inc., MA, USA) Wavelength:670 and 830 nm Channel:46	MSE of △oxyHb no difference
Li et al., 2020	16 (amnestic MCI)	67.6 ± 8.0	09:07	11.44 ± 3.92	MMSE:26.0 ± 2.5 MoCA:23.3 ± 3.9	Neuropsychological tests support daily living ability preserved and no other neurological and psychiatric disorders	Digit verbal span task	PFC, PC, OC.	Device:NIRx Medizintechnik GmbH, Germany Wavelength:760 and 850 nm Channel:46 Sampling rate:3.91 Hz	FC among PFC, PC and OC ↑
Niu et al., 2019	25 (amnestic MCI)	71.0 ± 8.1	13:12	10.6 ± 5.1	MMSE:23.5 ± 4.8 MOCA:19.0 ± 5.4 AVLT-I:6.3 ± 2.6 AVLT-D: 4.4 ± 3.9 AVLT-R:8.1 ± 3.6	Petersen’s	Resting state	PFC, TC, PC, OC.	Device:CW6 (Techen Co., Massachusetts) Wavelength:690 and 830 nm Channel:46 Sampling rate:50 Hz	Dynamic FC variability(The AUC of the power spectrum across the low frequency (<0.1 Hz) band) ↑ static FC no difference
Liu et al., 2023	54 (amnestic MCI)	66.8 ± 4.5	27:27	12.57 ± 2.84	MMSE:24.54 ± 2.58 MOCA:19.43 ± 3.32	Petersen’s	Delayed matching to sample task	PFC, TC, PC, OC.	Device:NirSmart-6000A Danyang Huichuang Medical Equipment Co., Ltd., China Wavelength:730 and 850 nm Channel:39	△oxyHb↓
Nakamura et al., 2021	28	71.0 ± 5.7	19:9	14.6 ± 2.0	MMSE:26.9 ± 2.1 CDRS:0.5 ± 0.4	Petersen’s	Modified serial number task	PFC, PC	Device:LABNIRS (Shimadzu Corporation; Kyoto, Japan) Channel:54	fNIRS index?
Nguyen et al., 2019	42	75.9 ± 3.6	NA	NA	NA	NIA-AA, IWG	N-back tasks	PFC	Device:Emitting diodes: OE-MV7385- P (Opto ENG; Korea) and photodiodes: Opto101 (Texas Instruments) Wavelength:730 and 850 nm Channel:6 Sampling rate:8 Hz	PFC FC no difference
Baik et al., 2022	22	73.6 ± 5.7	13:9	10.6 ± 2.3	K-MMSE:24.3 ± 5.0 K-MOCA:17.3 ± 6.5	Neuropsychological tests support daily living ability preserved and no other neurological and psychiatric disorders	N-back task Semantic VFT Stroop Resting state	PFC	Device:NIRSIT (OBELAB Inc., Seoul, Republic of Korea) Channel:48	N-back task:PFC △oxyHb no difference Semantic VFT:PFC △oxyHb ↓ Resting state:PFC △oxyHb no difference
Takahashi et al., 2022	61	78.2 ± 6.4	19:42	NA	MoCA:21.6 ± 3.1	Neuropsychological tests support daily living ability preserved and no other neurological and psychiatric disorders	Dual-task finger tapping with category VFT	PFC	Device:WOT100 (Hitachi HighTechnologies Corporation; Tokyo, Japan) Wavelength:705 and 830 nm Channel:10	PFC △oxyHb no difference
Talamonti et al., 2022	8	71.5 ± 4.1	7:1	13.3 ± 4.5	MoCA:24.1 ± 0.8	NIA-AA, Petersen’s	Dual-task talking with 2-back task	PFC, PC	Device:a portable fNIRS Wavelength:735 and 850 nm Channel:20 Channel:256	Baseline during single-task walking:ΔoxyHb ↑ dual-task walking:ΔoxyHb no difference
Ung et al., 2020	12	73.1 ± 8.2	8:4	primary 1 secondary 7 tertiary 4	MOCA:27.8 ± 1.9	Neuropsychological tests support daily living ability preserved and no other neurological and psychiatric disorders	Visuospatial working memory task with three levels of difficulty (level 1–3:low, medium, high)	PFC	Device:Multichannel continuous OT-R40 fNIRS system (Hitachi Medical Corporation, Japan) Wavelength:695 and 830 nm Channel:52 Sampling rate:10 Hz	Task difficulty level 1:slope of bilateral PFC △oxyHb no difference Task difficulty level 2–3:slope of bilateral PFC △oxyHb ↑
Vermeij et al., 2017	14	66.1 ± 3.9	10:4	13.3 ± 3.2	MMSE:27.1 ± 2.4	Petersen’s	N-back tasks with different working-memory load	PFC	Device:Oxymon Mk III (Artinis Medical System, The Netherlands) Wavelength:765,857 and 859 nm Channel:52 Sampling rate:125 Hz	0,1-back task :bilateral PFC △oxyHb↑ 2,3-back task:bilateral PFC △oxyHb no difference
Xu et al., 2024	21	71.2 ± 3.4	09:12	6.1 ± 3.3	MOCA:18.3 ± 3.0	Neuropsychological tests support daily living ability preserved and no other neurological and psychiatric disorders	standing balance;standing balance with sequence of subtracting 3 from a random number between 400 and 500	PFC	Device:portable near-infrared imaging system (LIGHTNIRS, Shimadzu Corp., Kyoto, Japan) Wavelength:780, 805, and 830 nm Channel:22 Sampling rate:13.3 Hz	Bilateral PFC △oxyHb↑
Yang et al., 2019	15	69.3 ± 7.1	01:14	11.2 ± 4.9	K-MMSE:25.1 ± 2.3	Neuropsychological tests support daily living ability preserved and no other neurological and psychiatric disorders	N-back task, Stroop task, VFT	PFC	Device:NIRSIT (OBELAB Inc., Rep. of Korea Wavelength: 780 and 850 nm Channel:48 Sampling rate:8.138 Hz	Left PFC △oxyHb↓
Yang et al., 2020	15	69.3 ± 7.1	01:14	11.2 ± 4.9	K-MMSE:25.1 ± 2.3	Neuropsychological tests support daily living ability preserved and no other neurological and psychiatric disorders	Semantic VFT	PFC	Device:NIRSIT (OBELAB Inc., Rep. of Korea Wavelength:780 and 850 nm Channel:48 Sampling rate:8.138 Hz	PFC △oxyHb↓
Yang and Hong, 2021	15	69.3 ± 7.1	01:14	11.2 ± 4.9	K-MMSE:25.1 ± 2.3	Neuropsychological tests support daily living ability preserved and no other neurological and psychiatric disorders	Resting state	PFC	Device:NIRSIT (OBELAB Inc., Rep. of Korea Wavelength:780 and 850 nm Channel:48 Sampling rate:8.138 Hz	PFC FC ↓
Yap et al., 2017	12	73.1 ± 8.2	08:04	primary 1 secondary 7 tertiary 4	MMSE:26.0 ± 3.1	neuropsychological tests support daily living ability preserved and no other neurological and psychiatric disorders	Semantic VFT	PFC, TC	Device:OT-R40 (Hitachi Medical Corporation, Japan) Channel:52 Sampling rate:10 Hz	PFC and TC △oxyHb no difference
Yeung et al., 2016a	26	69.2 ± 6.3	07:19	7.9 ± 4.5	CDRS, Chinese Version of the Mattis Dementia Rating Scale:149.8 ± 6.8	Petersen’s	N-back Task(0-back:low WM load;2-back high WM load)	PFC	Device: OEG-SpO2 system (Spectratech Inc., Tokyo, Japan) Wavelength:770 and 840 nm Channel:16 Sampling rate:12.21 Hz	0-back task:PFC bilateral △oxyHb no difference 2-back task:bilateral PFC △oxyHb↓
Yeung et al., 2016b	26	69.2 ± 6.3	07:19	7.9 ± 4.4	CDRS, Chinese Version of the Mattis Dementia Rating Scale:149.8 ± 6.8	NIA-AA, Petersen’s	Category fluency task	PFC	Device: OEG-SpO2 system (Spectratech Inc., Tokyo, Japan) Wavelength:770 and 840 nm Channel:16 Sampling rate:12.21 Hz	Bilateral PFC △oxyHb no difference No PFC lateralization
Yoo and Hong, 2019	15	NA	NA	NA	NA	Seoul Neuropsychological Screening Battery (Ahn et al., 2010)	Two-back task	PFC	Device: NIRSIT(OBELAB, Seoul, Korea) Wavelength:780 and 850 nm Channel:48 Sampling rate:8.138 Hz	Bilateral PFC △oxyHb↓
Yoon et al., 2019	15 (amnestic MCI 9)	amnestic: 66.9 ± 7.0 non-amnestic: 68.37 ± 6.54	NA	NA	NA	Seoul Neuropsychological Screening Battery	Semantic VFT Stroop task	PFC	Device: NIRSIT(OBELAB, Seoul, Korea) Channel:48	PFC △oxyHb no difference
Yu et al., 2020	23	63.2 ± 5.5	10:13	8.3 ± 3.9	MMSE:24.7 ± 1.2	Neuropsychological tests support daily living ability preserved and no other neurological and psychiatric disorders	Visuospatial working memory task	PFC	Device:NIRSIT (OBELAB, Seoul, South Korea) Wavelength:770 and 840 nm Channel:120 (52/68) Sampling rate:8.138 Hz	bilateral PFC △oxyHb↑ and PFC FC↑
Zeller et al., 2019	54	73.9 ± 1.8	29:25	NA	MMSE:24.7 ± 1.2	EADC	Resting state	PFC	ETG-4000 (Hitachi Medical; Tokyo, Japan) Wavelength:695 and 830 nm Channel:52 Sampling rate:10 Hz	PFC △oxyHb no difference
Zhang et al., 2022	64 (amnestic MCI)	67.5 ± 4.9	29:35	NA	MMSE:25.3 ± 2.43 MoCA:20.0 ± 1.9	2018 Guidelines for Diagnoses and Treatments of Dementias and Cognitive Impairments in China (Writing Group for Chinese Guidelines of Dementia and Cognitive Impairment Diagnoses and Treatments and Committee of Cognitive Disorders of Neurologist Branch of Chinese Medical Doctor Association, 2018) the Diagnostic and Statistical Manual of Mental Disorders—Fifth Edition (DSM-5) (American Psychiatric Association [APA], 2013)	Resting state	Whole-head channels	Device:NirSmart system (Danyang Huichuang Medical Equipment Corporation, China) Wavelength:730 and 850 nm Channel:71 Sampling rate:19 Hz	FC ↓
Zhang et al., 2023	64 (amnestic MCI)	75.2 ± 6.1	27:37	NA	NA	Neuropsychological tests support daily living ability preserved and no other neurological and psychiatric disorders	Stroop task	PFC, PC	Device:NirSmart system (Danyang Huichuang Medical Equipment Corporation, China) Wavelength:730 and 850 nm Channel:70 Sampling rate:11 Hz	PFC and PC delayed △oxyHb ↓

PFC, prefrontal cortex; MC, motor cortex; OC, occipital cortex; PC, parietal cortex; TC, temporal cortex; EC, effective connectivity; HSMG, High score intermediate group; LSMG, Low score intermediate group; △oxyHb, oxygenated hemoglobin concentration changes; MSE, multiscale entropy; FC, functional connectivity; VFT, verbal fluency task; EADC, MCI Working Group of the European Consortium on Alzheimer’s Disease; NINCDS-ADRDA, National Institute of Neurological and Communicative Disorders and Stroke-Alzheimer’s Disease and Related Disorders Association Work Group; NIA-AA, IWG, the National Institute on Aging and Alzheimer’s Association and International Working Group.

#### 3.2.2 Selection of task paradigms

Task paradigms are standard experimental procedures used for the classical experimental tasks in the study of cognitive psychology. Seven of the included studies adopted the N-back task (Nguyen et al., 2019; Baik et al., 2022; Talamonti et al., 2022; Yeung et al., 2016a; Yang et al., 2019; Yoo and Hong, 2019; Vermeij et al., 2017), a typical working memory task (WMT) paradigm. Other memory function–related task paradigms included Delayed Match to Sample Task, Digit Span Test, Visuospatial Working Memory Task, and Modified Serial Number Task (Khan et al., 2022; Kim et al., 2021; Li R. et al., 2018; Li et al., 2020; Liu et al., 2023; Nakamura et al., 2021; Ung et al., 2020; Yu et al., 2020). Moreover, tasks assessing thinking flexibility and planning, such as Shiritori and category fluency tasks and verbal fluency tasks(VFT), were also included (Kato et al., 2017; Katzorke et al., 2018; Baik et al., 2022; Takahashi et al., 2022; Yang et al., 2019; Yang et al., 2020; Yap et al., 2017; Yoon et al., 2019; Yeung et al., 2016b). Resting state, defined as subjects being awake, with eyes closed and relaxed, was used in 10 studies (Bu et al., 2019; Canario et al., 2022; Ghafoor et al., 2019; Ho et al., 2022; Li X. et al., 2018; Niu et al., 2019; Baik et al., 2022; Yang and Hong, 2021; Zeller et al., 2019; Zhang et al., 2022). The Stroop task, an attention-related task, was also chosen (Baik et al., 2022; Yang et al., 2019; Yoon et al., 2019; Zhang et al., 2023). Furthermore, three studies involved dual-task paradigms: finger tapping with category VFT, talking with the 2-back task, and balance control with sequential subtraction (Takahashi et al., 2022; Talamonti et al., 2022; Xu et al., 2024). Some studies also incorporated graded task difficulty and a combination of multiple tasks (Yeung et al., 2016a; Yang et al., 2019; Vermeij et al., 2017; Ung et al., 2020).

#### 3.2.3 Selection of ROIs

The frontal skull’s thinness and lack of hair interference facilitate fNIRS detection. Besides, the frontal cortex’s involvement in movement, cognition, and behavior regulation makes it a key area of focus. Therefore, the cerebral blood flow and cerebral oxygenation signals in the prefrontal cortex (PFC) were detected in all included studies. Furthermore, the detectors used in some studies covered multiple brain regions simultaneously to detect connections between brain regions, including functional connectivity (FC) and effective connectivity (EC).

#### 3.2.4 Selection of fNIRS device attributes

This systematic review revealed that fNIRS devices from different manufacturers were used in various studies. The number of channels varied from 6 to 204, and the maximum sampling rate was 138 Hz (3.91–125 Hz). Furthermore, the near-infrared light wavelengths detected in the studies varied from 600 to 900 nm.

#### 3.2.5 Selection of evaluation parameters

Functional near-infrared spectroscopy mainly reflects the changes in hemoglobin concentration in the ROI. Therefore, the changes in the concentration of oxygenated hemoglobin (△oxyHb) were the most commonly studied parameters in almost all included studies. Also, some studies analyzed the FC of ROIs (Bu et al., 2019; Canario et al., 2022; Ghafoor et al., 2019; Li et al., 2020; Nguyen et al., 2019; Yang and Hong, 2021; Yu et al., 2020; Zhang et al., 2022). The slope of △oxyHb was also a significant parameter (Ung et al., 2020; Li R. et al., 2018). Niu et al. (2019) selected dynamic FC variability as the research parameter, which was defined as the area under the curve of the power spectrum across the low-frequency (<0.1-Hz) band. Moreover, one study analyzed the multiscale entropy (MSE) of △oxyHb (Li X. et al., 2018). Lateralization, the asymmetric activation of brain regions, was also observed in some studies (Haberstumpf et al., 2022; Yeung et al., 2016b).

#### 3.2.6 Results of fNIRS

The change in oxyHb concentration during the task can reflect the activation level of ROIs. In this review, 10 studies suggested that the cortical △oxyHb of patients with MCI was reduced compared with that of the controls during the task (Haberstumpf et al., 2022; Kato et al., 2017; Khan et al., 2022; Liu et al., 2023; Baik et al., 2022; Yang et al., 2019; Yang et al., 2020; Yeung et al., 2016a; Yoo and Hong, 2019; Zhang et al., 2023), it was the most significant in the PFC. The PFC activation of patients with MCI was normal under a low WMT load, which showed decreased activation with an increase in the WMT load (Yeung et al., 2016a). Patients with MCI having lower cognitive scores were more likely to show reduced PFC activation (Kato et al., 2017). Baik et al. (2022) found that △oxyHb in the PFC was reduced in the semantic VFT but not in the N-back task or resting state. In addition, a study compared the bilateral PFC activation of patients with MCI and found that the activation reduction of the left PFC was more significant (Yang et al., 2019). Moreover, the EC and FC in patients with MCI was also reduced (Bu et al., 2019; Ghafoor et al., 2019; Yang and Hong, 2021; Zhang et al., 2022). The slope of △oxyHb decreased in patients with MCI, indicating reduced activation of the PFC (Li R. et al., 2018).

However, some other studies reported opposite results, demonstrating higher PFC activation in patients with MCI than in controls (Kim et al., 2021; Li et al., 2020; Talamonti et al., 2022; Ung et al., 2020; Vermeij et al., 2017; Xu et al., 2024; Yu et al., 2020). The slope of FPC △oxyHb in patients with MCI increased more significantly under a higher WMT load than a lower WMT load (Ung et al., 2020). However, a higher WMT load did not cause an increase in FPC △oxyHb compared with a lower WMT load (Vermeij et al., 2017). Talamonti et al. (2022) also suggested that △oxyHb increased during single-task walking but not during dual-task walking.

Furthermore, nine studies did not obtain a difference in △oxyHb and FC between patients with MCI and healthy controls (Canario et al., 2022; Ho et al., 2022; Li R. et al., 2018; Nguyen et al., 2019; Takahashi et al., 2022; Yap et al., 2017; Yeung et al., 2016b; Yoon et al., 2019; Zeller et al., 2019). Some studies selected other research fNIRS parameters. Li X. et al. (2018) found no difference in the MSE of △oxyHb between patients with MCI and healthy controls. Niu et al. (2019) suggested that the static FC did not change, but dynamic FC variability increased in MCI. Besides, the △oxyHb of the left PFC in patients with MCI did not increase in the same manner as in a healthy population, which was defined as frontal lateralization (Haberstumpf et al., 2022; Yeung et al., 2016b).

## 4 Discussion

This systematic review explored the differences in fNIRS between elderly patients with MCI and healthy controls, revealing mixed results. Some studies suggested that cortical activation was reduced in MCI, while others reported opposite results. Additionally, some studies reached neutral conclusions.

The aforementioned results were mixed, which might have been caused by the heterogeneity of the MCI group and the lack of standardization in methods (Stern et al., 2012; Butters et al., 2023). MCI is difficult to diagnose and classify into subtypes (Díaz-Mardomingo et al., 2017), leading to inevitable heterogeneity. The difference in task types, ROIs, and monitoring time make it difficult to standardize methods. According to cognitive load theory and scaffolding theory of aging and cognition, the PFC can perform compensatory activation to offset cognitive deterioration in MCI (Park and Bischof, 2013; Merlo et al., 2019). This theory might partially explain the differences in ROI activation under the aforementioned heterogeneous conditions. This compensation did not occur at low WMT load or during the resting state, which is why no differences in ROI activation were noted between patients with MCI and healthy population at that time (Baik et al., 2022; Ung et al., 2020; Yeung et al., 2016a). When the WMT load appropriately increases, compensation activates in patients with MCI but not in healthy subjects (Ung et al., 2020). However, such compensation is limited. Therefore, as the WMT load further increases, patients with MCI show reduced activation of ROIs (Baik et al., 2022; Yeung et al., 2016a). Besides, we speculate that this limitation might be common in aging elderly subjects during excessive WMT load (Ren et al., 2012; Yap et al., 2017; Heinzel et al., 2014). At this point, the activation inertness of ROIs between the MCI group and the control group might become similar (Talamonti et al., 2022; Vermeij et al., 2017). The FC between PFC and other brain regions was reduced in MCI (Farràs-Permanyer et al., 2015; Cohen et al., 2014). However, the FC within the PFC was activated through a compensatory mechanism (Qi et al., 2010; Liang et al., 2014). In the included studies, we found that FC was mostly reduced in the resting state (Bu et al., 2019; Ghafoor et al., 2019; Yang and Hong, 2021; Zhang et al., 2022) and increased during tasks (Li et al., 2020; Yu et al., 2020). We speculated that this might be related to more compensatory function assistance between brain regions in task states. Moreover, the neural compensatory mechanisms may involve upregulation in excitatory synapses, synaptic and metabolic activity, enhancement of neurotrophic milieu, and changes in glial cell reactivity and inflammation (Merlo et al., 2019). This enhanced synaptic activity thus manifests in the form of hyperactivation in the affected region (Merlo et al., 2019). However, such neural compensation may not be timely. Patients with MCI may require increased oxygen consumption due to functional impairments. This leads to a greater reduction in oxyHb shortly after the cognitive task onset, followed by the delayed oxyHb influx. This might explain the decrease in △oxyHb in some studies, which might be due to the short fNIRS monitoring time. In addition, the cognitive task selectively activated the left PFC, leading to the difference in △oxyHb in bilateral PFC (Arai et al., 2006).

Although these differences may be partly attributed to the lens of cognitive load theory and the scaffolding theory of aging and cognition, we cannot ignore the limitations of current fNIRS studies in MCI. Since most current studies do not specify MCI subtypes based on Petersen and/or DSM criteria, we are unable to provide a more detailed interpretation of the results according to subtype classifications. Furthermore, these limitations encompass the lack of standardization in task difficulty, task types, and monitoring duration for different patients with MCI.

## 5 Conclusion

Progressive studies on fNIRS in elderly patients with MCI have been conducted using various task paradigms, evaluation parameters, and ROIs to provide an objective marker for MCI diagnosis. However, the current research findings are inconsistent. To obtain consistent, accurate, and reliable evidence for the application of fNIRS in MCI, future studies should clearly characterize the type of MCI the participants have according to Petersen and/or DSM criteria. And representative ROIs and suitable task paradigms for various patients with MCI need be aimed. Additionally, the duration of fNIRS monitoring should be further explored. Larger sample sizes and more appropriate research indicators are also essential. Prospective studies should be encouraged to evaluate the potential prognostic role of fNIRS. Furthermore, comparing NIRS findings with objective measurements (e.g., AD biomarkers) would allow for the estimation of sensitivity and specificity of the method for dementia diagnosis.

## Data Availability

The original contributions presented in this study are included in this article/Supplementary material, further inquiries can be directed to the corresponding author.
