# Supplementary material for: Identification of functional near-infrared spectroscopy for older adults with mild cognitive impairment: a systematic review
Source: Front Aging Neurosci. 2025 Apr 9;17:1492800. doi: 10.3389/fnagi.2025.1492800 (PMC12014625; doi:10.3389/fnagi.2025.1492800)
Supplement: Supplementary file 1 [file Table_1.DOCX]

PubMed

#1 ((((mild cognitive impairment[Title/Abstract])) OR (mild cognitive dysfunction[Title/Abstract])) OR (mild cognitive disorder[Title/Abstract])) OR (MCI[Title/Abstract])

#2 ((((((( fNIRS[Title/Abstract])) OR (functional near infrared spectroscopy[Title/Abstract])) OR (functional near-infrared spectroscopy[Title/Abstract])) OR (NIRS[Title/Abstract])) OR (near-infrared spectroscopy[Title/Abstract])) OR (near infrared spectroscopy[Title/Abstract])) OR (Spectroscopy, Near-Infrared[Mesh])

#3 #1and#2

EMBASE

Embase Session Results

.......................................................

No. Query Results Results Date

#13. #5 AND #12 180 15 Jun 2024

#12. #6 OR #7 OR #8 OR #9 OR #10 OR #11 23,433 15 Jun 2024

#11. 'near infrared spectroscopy':ab,ti 19,298 15 Jun 2024

#10. 'near-infrared spectroscopy':ab,ti 19,299 15 Jun 2024

#9. 'nirs':ab,ti 10,920 15 Jun 2024

#8. 'functional near infrared spectroscopy':ab,ti 4,185 15 Jun 2024

#7. 'fnirs':ab,ti 3,860 15 Jun 2024

#6. 'functional near-infrared spectroscopy'/exp OR 4,922 15 Jun 2024

'functional near-infrared spectroscopy':ab,ti

#5. #1 OR #2 OR #3 OR #4 69,382 15 Jun 2024

#4. 'mci':ab,ti 44,037 15 Jun 2024

#3. 'mild cognitive disorder':ab,ti 137 15 Jun 2024

#2. 'mild cognitive dysfunction':ab,ti 285 15 Jun 2024

#1. 'mild cognitive impairment'/exp OR 'mild 49,929 15 Jun 2024

cognitive impairment':ab,ti

Web of Science

#1 TS=((fNIRS) OR (functional near infrared spectroscopy) OR (Functional Near-Infrared spectroscopy) OR (NIRS) OR (near-infrared spectroscopy))

#2 TS=((mild cognitive impairment) OR (mild cognitive dysfunction) OR (mild cognitive disorder) OR (MCI) )

#3 #1 and #2

Cochrane Library

#1 (mild cognitive impairment):ti,ab,kw

#2 (mild cognitive dysfunction):ti,ab,kw

#3 (mild cognitive disorder):ti,ab,kw

#4 (MCI):ti,ab,kw

#5 #1 OR #2 OR #3 OR #4 OR

#6 (functional near infrared spectroscopy):ti,ab,kw

#7 (functional near-infrared spectroscopy):ti,ab,kw

#8 (near infrared spectroscopy):ti,ab,kw

#9 (near-infrared spectroscopy):ti,ab,kw

#10 (fNIRS):ti,ab,kw

#11 (NIRS):ti,ab,kw 1514

#12 #6 OR #7 OR #8 OR #9 OR #10 OR #11

#13 #5 AND #12
